# New Types of Magnetic Nanoparticles for Stimuli‐Responsive Theranostic Nanoplatforms

**DOI:** 10.1002/advs.202305459

**Published:** 2023-11-21

**Authors:** Shuren Wang, Yanglong Hou

**Affiliations:** ^1^ Beijing Key Laboratory for Magnetoelectric Materials and Devices School of Materials Science and Engineering Peking University Beijing 100871 China; ^2^ School of Materials Sun Yat‐Sen University Shenzhen 518107 China

**Keywords:** immunotherapy, magnetic nanoparticles, nanozymes, stimuli‐responsive treatments, theranostics

## Abstract

Magnetic nanomaterials have played a crucial role in promoting the application of nanotechnology in the biomedical field. Although conventional magnetic nanomaterials such as iron oxide nanoparticles (NPs) are used as biosensors, drug delivery vehicles, diagnostic and treatment agents for several diseases, the persistent pursuit of high‐performance technologies has prompted researchers to continuously develop new types of magnetic nanomaterials such as iron carbide NPs. Considering their potential application in biomedicine, magnetic NPs responsive to exogenous or endogenous stimuli are developed, thereby enhancing their applicability in more complex versatile scenarios. In this review, the synthesis and surface modification of magnetic NPs are focused, particularly iron carbide NPs. Subsequently, exogenous and endogenous stimuli‐responsive magnetic NP‐based theranostic platforms are introduced, particularly focusing on nanozyme‐based technologies and magnetic NP‐mediated immunotherapy, which are emerging stimuli‐responsive treatments. Finally, the challenges and perspectives of magnetic NPs to accelerate future research in this field are discussed.

## Graphical abstract

Magnetic NPs with theranostic function are designed to respond to exogenous or endogenous stimuli, thereby making them versatile and adaptable to more complex biomedical application scenarios. Endogenous stimuli include acidity, hydrogen peroxide, and intrinsic immune responses. The exogenous stimuli include light, magnetic field, etc.

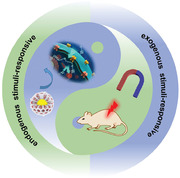

## 1 Introduction

Magnetic nanomaterials have been extensively exploited in various fields that are closely related to our daily lives,^[^ ^1^ ^]^ such as catalysis,^[^ ^2^ ^]^ electromagnetic wave adsorption,^[^ ^3^ ^]^ and especially biomedicine.^[^ ^4^ ^]^ Conventional magnetic nanomaterials contain magnetic elements (e.g., iron, cobalt, nickel, and manganese), and their physical and chemical properties are usually optimized by adjusting their sizes, shapes, structures, and chemical components to improve their utility in biomedicine. Considering their low toxicity and good biocompatibility, iron oxide nanoparticles (NPs), especially Fe_3_O_4_ NPs, are the most prevalent nanomaterials,^[^ ^5^ ^]^ and have significantly contributed to the development of magnetic resonance imaging (MRI),^[^ ^6^ ^]^ biosensing,^[^ ^7^ ^]^ and drug delivery,^[^ ^4b^ ^]^ among other applications. However, the bare iron oxide NPs are often easily degraded upon direct exposure to the environment, resulting in poor stability and dispersity. More importantly, several inherent bottlenecks prevent the improvement of the performance of iron oxide NPs, despite attempts to modify their size, morphology, and other features. For example, the magnetic properties of iron oxide NPs are not sufficiently good to achieve MRI with high sensitivity. The photothermal conversion performance of iron oxide NPs also warrants further improvement, to meet the widespread demand for photothermal therapy (PTT).^[^ ^4a^ ^]^ Hence, it is crucial to develop new types of magnetic nanomaterials to expand their application in the biomedical field.

Owing to the ongoing research in this field, several new types of magnetic nanomaterials have emerged that possess superior physical and chemical properties compared with the conventional magnetic nanomaterials. In 2012, Hou et al. proposed a universal and controllable method for the synthesis of iron carbide NPs,^[^ ^8^ ^]^ which has garnered great interest from researchers in the interdisciplinary field of biomedicine and materials science. The precise control of the crystal structures of iron carbide NPs is achieved by modulating the selectively adsorbed halide ions. Meanwhile, iron carbide NPs are also endowed with controllable ultrasmall sizes and desirable stability. As an emerging magnetic nanomaterial, iron carbide NPs contain both carbon and iron elements, thus exhibiting several excellent and unique properties, such as high saturation magnetization (≈140 emu g^−1^), strong corrosion resistance, excellent photothermal conversion performance, and good catalytic performance. Together, these properties make iron carbide NPs stand out in the field of cancer theranostics. For instance, a strong magnetic performance allows the use of iron carbide NPs in MRI,^[^ ^9^ ^]^ magnetic targeting, and magnetic separation^[^ ^10^ ^]^; moreover, their prominent catalytic performance can trigger iron carbide NPs to generate toxic hydroxyl radicals (·OH) for chemodynamic therapy (CDT).^[^ ^11^ ^]^ Additionally, heat‐generating properties can further expand the application of iron carbide NPs in hyperthermia, such as magnetic hyperthermia (MHT)^[^ ^12^ ^]^ and PTT.^[^ ^13^ ^]^

Considering the multifunctional combination of imaging and treatment afforded by iron carbide NPs, these materials can act as strong candidates for cancer theranostic platforms. Meanwhile, the theranostic function of iron carbide NPs can be also further enhanced by the introduction of exogenous or endogenous stimuli.^[^ ^14^ ^]^ For instance, light is a manageable stimulus that can be used to provide PTT and photodynamic therapy (PDT).^[^ ^15^ ^]^ Flexible MHT with different degrees at fixed locations can be achieved by adjusting the position and strength of the magnetic field.^[^ ^16^ ^]^ In addition to exogenous stimuli, endogenous stimuli are widely used to provide controllable treatments. For instance, the tumor microenvironment (TME) exhibits different characteristics compared with normal tissues, such as hypoxia,^[^ ^17^ ^]^ weak acidity,^[^ ^18^ ^]^ high hydrogen peroxide(H_2_O_2_) content,^[^ ^19^ ^]^ high glutathione (GSH) level,^[^ ^20^ ^]^ and low catalase activity.^[^ ^21^ ^]^ These distinct physiological features can be used as internal stimuli for cancer‐specific theranostics, such as CDT. Using these internal stimuli, iron carbide NPs can undergo specific reactions at the tumor sites, increasing the precision of the treatment and further enhancing their biosafety in vivo.

**Figure 1:**
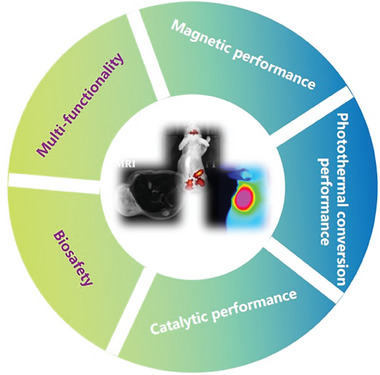
Schematic of the advantages of iron carbide NPs based on their intrinsic physical and chemical properties and application scenarios.

Iron carbide NPs can respond to exogenous or endogenous stimuli and are defined as new types of magnetic NPs with better biocompatibility, higher imaging accuracy, and stronger therapeutic effects than their counterparts (**Figure** **1**). In this review, according to different stimuli, we introduce the various synthesis methods and surface modifications available for iron carbide NPs, including single‐phase iron carbide NPs and composite‐phase iron carbide NPs; furthermore, we emphasize the key factors for designing nanostructures based on the biological functions of each part of the system. Subsequently, we summarize the existing exogenous or endogenous stimulus‐responsive theranostic platforms based on magnetic NPs, particularly focusing on the iron carbide NP‐based nanozymes and magnetic NP‐mediated immunotherapy. Finally, we discuss the challenges and perspectives in this field to facilitate future investigations.

## 2 Synthesis and Modification of Stimulus‐Responsive Magnetic NPs

As iron carbide NPs are a new type of magnetic NPs, their controlled synthesis is crucial for the development of the application field of magnetic materials. Therefore, in this section, we focus on the different synthesis methods and surface modifications of iron carbide NPs, rather than those of other magnetic NPs. Methods used for the synthesis of iron carbide NPs can be broadly categorized as physical and chemical approaches.^[^ ^22^ ^]^ Physical approaches include physical vapor deposition, plasma, and laser methods, which can provide more defined and chemically pure structures, albeit with a limited variety. Conversely, by adjusting the synthetic conditions, a broader range of iron carbide NPs can be obtained through the use of more versatile chemical methods, such as the sol−gel process and high‐temperature organic phase methods, which were described in detail in our previous review.^[^ ^22^ ^]^ In this section, we focus on the selected strategies for the design of iron carbide NPs in different phases (i.e., single‐phase and composite‐phase iron carbide NPs) and their specific biomedical applications.

### 2.1 Single‐Phase Stimulus‐Responsive Iron Carbide NPs

**Figure 2:**
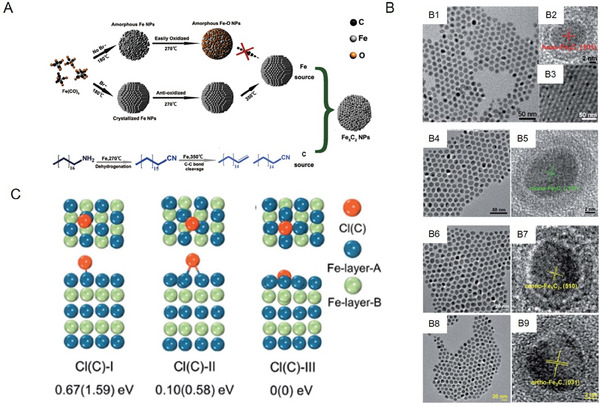
A) Schematic of the mechanism of formation of Fe_5_C_2_ NPs. Reproduced with permission.^[^ ^8^ ^]^ Copyright 2012 American Chemical Society. B) Transmission electron microscopy (TEM) and high‐resolution TEM (HRTEM) images of hexa‐Fe_2_C NPs (B1‐B3), mono‐Fe_2_C NPs (B4‐B5), mono‐Fe_5_C_2_ NPs (B6‐B7), and ortho‐Fe_3_C NPs (B8‐B9). C) Density functional theory (DFT) simulation of three adsorption configurations of a Cl (C) atom on the surface of Fe (101). Reproduced with permission.^[^ ^23^ ^]^ Copyright 2017 Royal Society of Chemistry.

Previous studies have focused on developing methods for the controlled synthesis of single‐phase iron carbide NPs with good monodispersity and stability, which have been the focus and roadblock of research in this field. In 2012, Hou et al. synthesized iron carbide NPs (Fe_5_C_2_) via a facile wet‐chemical route at relatively mild conditions (623 K, 0.1 MPa) and found that bromide was the key agent for inducing the conversion of Fe(CO)_5_ to Fe_5_C_2_ in the synthetic process; bromine ions enhanced the stability of Fe_5_C_2_ NPs by protecting them from oxidation during the carbonization process^[^ ^8^ ^]^ (**Figure** **2**A). Subsequently, Hou et al. provided a versatile chemical route toward the synthesis of iron carbide NPs with different phases, including hexagonal Fe_2_C and monoclinic syngony Fe_2_C, as well as monoclinic syngony Fe_5_C_2_ and orthorhombic syngony Fe_3_C (Figure 2B).^[^ ^23^ ^]^ As demonstrated by density functional theory (DFT) simulations, this study clarified the mechanism underlying the Cl ion‐mediated regulation of the phase of iron carbide NPs. Bonding energy between the Fe─C and Fe─Cl atoms indicated that the bond between Fe and C atoms was weakened by the selective adsorption of Cl, leading to the formation of Fe_5_C_2_ and Fe_3_C NPs with a lower carbon content by reducing the penetration of C atoms (Figure 2C).^[^ ^23^ ^]^

To ensure in vivo biosafety, it is essential to introduce surface modifications onto iron carbide NPs. Usually, hydrophilic chemicals such as 1,2‐distearoyl‐sn‐glycerol‐3‐phosphoethanolamine‐N‐[amino(polyethylene glycol)−2000] (DSPE‐PEG‐NH_2_)^[^ ^13^ ^]^ and bovine serum albumin (BSA)^[^ ^24^ ^]^ are common candidates for surface modification. Surface modification of iron carbide NPs with these chemicals can enhance their hydrophilicity and biocompatibility in vivo, thereby reducing their biological toxicity and extending their half‐life in the blood, and facilitating their subsequent biological applications. Moreover, they are also convenient for connecting multiple functional molecules, as chemical modification can provide abundant surface groups. For example, the representative imaging agent indocyanine green (ICG)^[^ ^25^ ^]^ is often conjugated on the surface of iron carbide NPs for multimodal imaging involving near‐infrared (NIR) II fluorescence imaging.^[^ ^26^ ^]^ Several specific peptides that target cancer cells, such as HER2 receptors (Z_HER2:342_)^[^ ^13^ ^]^ and the tumor‐homing penetration peptide iRGD,^[^ ^27^ ^]^ are also coated to promote the enrichment of iron carbide NPs at tumor sites, thus improving their diagnostic and therapeutic effects.

### 2.2 Composite‐Phase Stimulus‐Responsive Iron Carbide NPs

**Figure 3:**
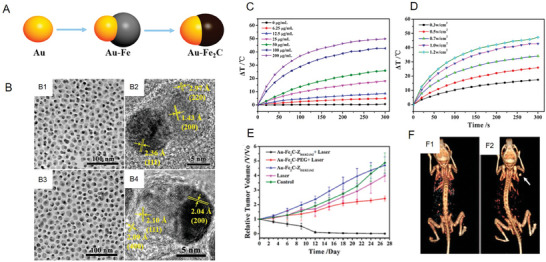
A) Schematic of the synthesis of Au–Fe_2_C Janus NPs. B) TEM and HRTEM images of Au–Fe Janus NPs (B1‐B2) and Au–Fe_2_C Janus NPs (B3‐B4). C) Temperature changing curves of Au–Fe_2_C Janus NPs at different concentrations under NIR laser irradiation (λ = 808 nm, 1 W cm^−2^). D) Temperature changing curves of Au–Fe_2_C Janus NPs (100 µg mL^−1^) under NIR laser irradiation (λ = 808 nm) with different laser power densities. E) Tumor relative volume curve of tumor‐bearing mice that received different treatments. F) 3D‐reconstructed CT images acquired before (F1) and after (F2) the intratumor injection of Au–Fe_2_C Janus NPs. Reproduced with permission.^[^ ^30^ ^]^ Copyright 2017, American Chemical Society.

In the complex and changeable TME, iron carbide NPs in a single phase generally cannot provide precise diagnosis and treatment. Therefore, Hou et al. also aimed to develop a series of new composite‐phase iron carbide NPs for expanding their theranostic applications. For instance, gold‐based NPs have remarkable photothermal conversion performance and can mediate computed tomography (CT) imaging, which indicates their suitability as a component of nanocomposites.^[^ ^28^ ^]^ Because of their outstanding optical properties, Au–Fe_3_O_4_ NPs exhibit multiple functions in cancer theranostics.^[^ ^29^ ^]^ Based on the design concept described above, Hou et al. developed a monodisperse type of Au–Fe_2_C Janus NPs. First, Au–Fe_2_C Janus NPs were synthesized using the following steps: Au seed preparation, Au–Fe heterostructure formation, and Au–Fe_2_C Janus NP carburization (**Figure** **3**A). The TEM results indicated that both Au–Fe Janus NPs and Au–Fe_2_C Janus NPs had remarkable monodispersity, which was beneficial for subsequent theranostic applications (Figure 3B). One major advantage of Au–Fe_2_C Janus NPs was that they exhibited a wide absorption band in the NIR region and high photothermal conversion efficiency (30.2%) in vitro, indicating that they were excellent PTT agents (Figure 3C,D). When Au–Fe_2_C Janus NPs were enriched at the tumor sites, the temperature of the tumor center exceeded 42 °C under laser irradiation, which tremendously promoted the inhibitory effect on tumor growth (Figure 3E). Meanwhile, because of the addition of Au components, Au–Fe_2_C Janus NPs finally realized triple‐modal imaging including MRI, multispectral photoacoustic tomography (MSOT), and CT, resulting in a more accurate diagnosis of tumor lesion tissues (Figure 3F).^[^ ^30^ ^]^ In addition to Au, other functional elements (e.g., Cu, Ag, etc.) have also been introduced into single‐phase iron carbide NPs and have been confirmed to further improve their physicochemical properties, which will be discussed in the next section.

## 3 Exogenous Stimulus‐Responsive Theranostic Nanoplatforms in Cancer Therapy

### 3.1 Light‐Responsive Theranostic Nanoplatforms

**Figure 4:**
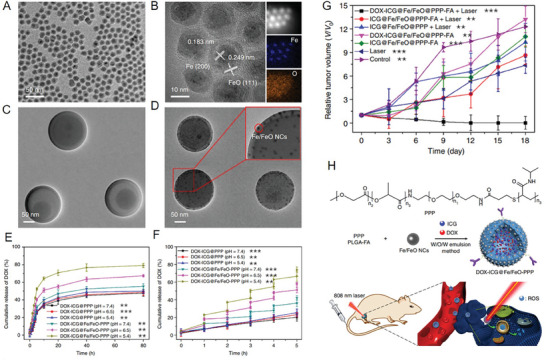
A) TEM image of Fe/FeO NPs. B) HRTEM image of Fe/FeO NPs. C) TEM image of DOX‐ICG@PPP nanocapsules. D) TEM image of DOX‐ICG@Fe/FeO‐PPP nanocapsules. E) Release curve of DOX from DOX‐ICG@PPP nanocapsules and DOX‐ICG@Fe/FeO‐PPP nanocapsules at different pH values (7.4, 6.5, and 5.4) at 37°C. F) Release curve of DOX from DOX‐ICG@PPP nanocapsules (pH 7.4, 6.5, and 5.4) and DOX‐ICG@Fe/FeO‐PPP nanocapsules (pH 7.4, 6.5, and 5.4) using four laser on/off cycles under laser irradiation (808 nm, 0.3 W cm^−2^, 5 min). G) Tumor relative volume curve of tumor‐bearing mice that received different treatments. H) Schematic of the synthetic process and biomedical application of DOX‐ICG@Fe/FeO‐PPP nanocapsules. Reproduced with permission.^[^ ^36^ ^]^ Copyright the Nature Publishing Group.

Light is a common and flexible exogenous stimulus, and related nanotechnology‐mediated treatments include PTT and PDT. The key therapeutic mechanism underlying this type of therapy comprises the conversion of the absorbed energy to heat energy or chemical energy, eventually inhibiting or eliminating tumor cells.^[^ ^31^ ^]^ More specifically, PTT relies on the intrinsic photothermal properties of NPs or photosensitive agents to release vibrational energy via visible and NIR light.^[^ ^32^ ^]^ Compared with other treatments, PTT can optimize the precision of treatments via the targeted enrichment of NPs at tumor sites and adjustment of light intensity, while using the strong penetration of NIR light to treat deep tumors.^[^ ^33^ ^]^ Diversified magnetic NPs with good photothermal properties have been developed for PTT, such as Fe@Fe_3_O_4_ NPs^[^ ^34^ ^]^ and ultrasmall (<10 nm) Fe_3_O_4_@Cu_2‐x_S NPs.^[^ ^35^ ^]^ Hou et al. also developed iron oxide NPs with core–shell nanostructures, i.e., Fe/FeO NPs encapsulated with ICG and doxorubicin (DOX), a chemotherapy drug, in PLGA‐PEG‐PNIPAM (PPP), a derivative of temperature‐sensitive poly (lactic acid–glycolic acid) copolymer (**Figure** **4**A–D). In this study, the ingenious design induced the controlled degradation of PPP using the photothermal effect of Fe/FeO NPs and the weak acidity of the TME, ultimately releasing the Fe/FeO NPs, ICG, and DOX at the tumor sites (Figure 4E,F). This strategy based on synergistic therapy afforded a remarkable therapeutic effect on oral epithelial cancer in vivo through chemotherapy, PTT, and CDT (Figure 4G,H).^[^ ^36^ ^]^

**Figure 5:**
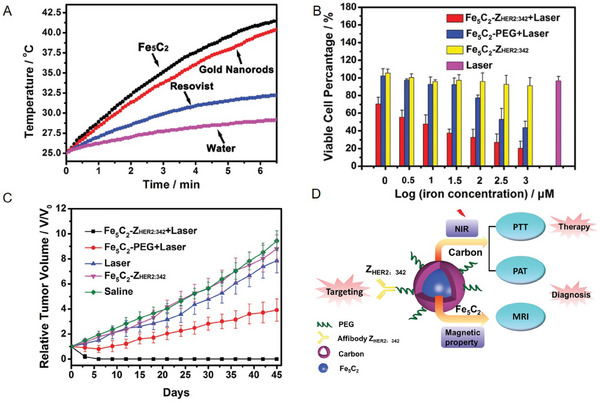
A) Temperature changing curves of Resovist/gold nanorods/Fe_5_C_2_ NPs in aqueous dispersions at the same concentration (0.5 mm Fe or Au) under NIR laser irradiation (λ = 808 nm, 2 W cm^−2^). B) Viability test of SK‐OV‐3 cells after different treatments. C) Tumor relative volume curve of tumor‐bearing mice that received different treatments. D) Schematic of the synthesis of Fe_5_C_2_ NPs as a theranostic nanoplatform. Reproduced with permission.^[^ ^13^ ^]^ Copyright 2014, Wiley‐VCH.

Moreover, Hou et al. have conducted a systematic exploration of the PTT applications of iron carbide NPs. They were initially recognized for their outstanding photothermal conversion performance, which had tremendous therapeutic implications. Previous studies found that Fe_5_C_2_ NPs induced a greater temperature increment than other agents, such as gold nanorods and Resovist under NIR laser irradiation (λ = 808 nm, 2 W cm^2^); this indicated that Fe_5_C_2_ NPs had better photothermal properties compared with other materials (**Figure** **5**A). To achieve active targeting of tumor cells, Z_HER2:342_ was attached to the surface of Fe_5_C_2_ NPs. HER2 receptors were overexpressed on the cytomembrane of several specific tumor cells; thus, Z_HER2:342_‐conjugated Fe_5_C_2_ NPs accumulated in abundance at the tumor sites via interactions between proteins. As shown in Figure 5B,C, Fe_5_C_2_ NPs exhibited an adequate tumor‐inhibition effect under exogenous laser irradiation at the cellular and animal levels, confirming their potential as PTT agents. In addition, a new cancer theranostic strategy has also been proposed based on MRI/photoacoustic tomography (PAT)‐guided PTT, which exploited the versatility of Fe_5_C_2_ NPs for imaging. This dual‐modal imaging not only improved the sensitivity of tumor diagnosis but also allowed the detection of tumor boundaries in a clearer manner, which was conducive to the early diagnosis of tumors (Figure 5D).^[^ ^13^ ^]^ To improve PTT‐based treatment strategies using iron carbide NPs, Hou et al. designed a multistimulus‐controlled drug carrier by coating Fe_5_C_2_ NPs with BSA. When exposed to NIR light or acidic conditions, this carrier could degrade and release DOX. This highlights the great potential of Fe_5_C_2_ NPs as a remote‐controlled platform for photochemothermal cancer therapy.^[^ ^24a^ ^]^

In addition to PTT, PDT is a type of treatment that requires light stimulation. Moreover, the presence of oxygen and a photosensitizer is equally necessary to generate reactive oxygen species (ROS) for killing cancer cells.^[^ ^15^, ^37^ ^]^ The modulation of spatiotemporal delivery of light can help in administering PDT at the tumor sites rather than in normal tissues, minimizing the toxic side effects of chemotherapy. Some conventional magnetic NPs such as FeS_2_^[^ ^38^ ^]^ NPs can function as PDT agents,^[^ ^39^ ^]^ and the modification of the surface of magnetic NPs using photosensitizers is a common strategy to enhance their effectiveness in PDT.^[^ ^40^ ^]^ However, relevant research on iron carbide NPs has not been reported and is worthy of future investigation.

### 3.2 Magnetic Field‐Responsive Theranostic Nanoplatforms

Magnetic fields are another common external stimulus that can mediate MHT. MHT causes irreversible damage to cancer cells by inducing increased temperatures (up to 39–45 °C) in the center of tumor tissues. The characteristics of magnetic NPs, such as their size, shape, structure, and magnetic properties, have a complex influence on the effectiveness of MHT. An important index to evaluate the efficiency of heat generation is the specific absorption rate (SAR) or specific loss power, which is usually affected by the frequency and magnitude of the ambient alternating magnetic field and intrinsic properties of the magnetic NPs. High SAR and heat production efficiency are often sought in the biomedical field because they can reduce the doses of injected magnetic NPs in vivo.^[^ ^41^ ^]^ Superparamagnetic Fe_3_O_4_ NPs are most widely used for MHT because of their strong magnetic properties and low biotoxicity^[^ ^42^ ^]^ and composite‐phase Fe_3_O_4_ NPs with core–shell heterostructures, such as Fe_3_O_4_@CoFe_2_O_4_^[^ ^43^ ^]^ and FeO@Fe_3_O_4_,^[^ ^44^ ^]^ have been proven to have higher values of SAR. Moreover, some iron carbide NPs, such as Fe_2.2_C,^[^ ^12^, ^45^ ^]^ have gradually gained prominence in the field of MHT because of their excellent heat‐generating efficiency. Considering the need for good biocompatibility in in vivo treatments, Chaudret et al. prepared highly magnetic 15 nm iron carbide NPs in aqueous media. The desired water solubility and colloidal stability of Fe_2.2_C NPs were achieved by designing and using specific dopamine‐based ligands, with Fe_2.2_C NPs displaying high SARs in water/glycerol mixture media (SAR up to 1000 W g^−1^ in water at 100 kHz, 47 mT), which suggests a tremendous application potential for iron carbide NPs in MHT.^[^ ^45^ ^]^

However, MHT can easily cause an inevitable technical bottleneck, i.e., heating of the tumor region without impairing normal tissues. Hence, this poses a challenge for the application of magnetic NPs in MHT. One familiar strategy in this context is the implantation of magnetic NPs in tumor tissues at designated locations and the implementation of multiple MHTs to improve the utility of magnetic NPs and reduce unnecessary damage caused by multiple injections of magnetic NPs. One example of this approach is the loading of magnetic NPs onto nanocapsules modified with poly(organophosphazene) (PPZ) hydrogels. Polymeric nanocapsules were self‐assembled using an amphiphilic and thermosensitive system. PPZ existed as a hydrogel below 37 °C for more than 3 weeks within tumor tissues, retaining magnetic NPs for an extended period, which ensured the possibility of administering multiple MHTs without damaging the surrounding healthy tissues.^[^ ^46^ ^]^

## 4 Endogenous Stimulus‐Responsive Theranostic Nanoplatforms for Cancer Therapy

### 4.1 Acidity‐Responsive Theranostic Nanoplatforms

**Figure 6:**
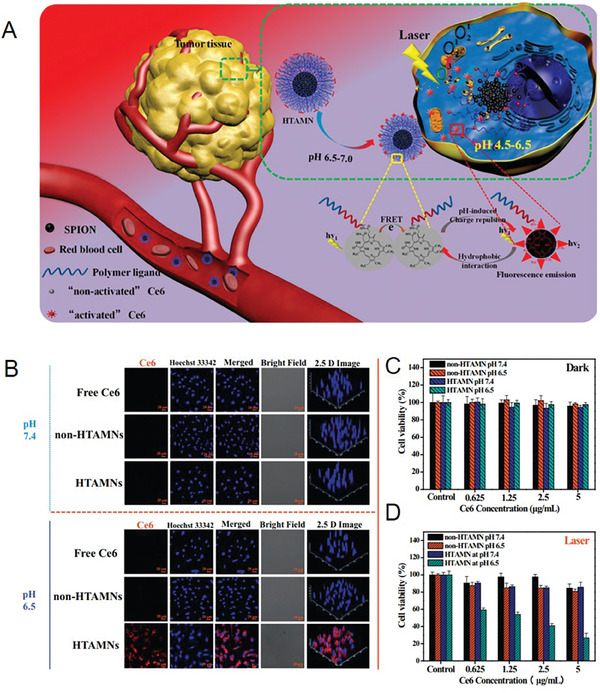
A) Schematic of the theranostic mechanism of HTAMNs. B) Confocal images of the cellular uptake of HepG2 cancer cells that received different treatments. C) Phototoxicity of HTAMNs or non‐HTAMNs against HepG2 cancer cells at pH 7.4 or 6.5 without laser irradiation, as assessed using an MTT assay. D) Phototoxicity of HTAMNs or non‐HTAMNs against HepG2 cancer cells at pH 7.4 or 6.5 with laser irradiation, as assessed using an MTT assay. Reproduced with permission.^[^ ^48^ ^]^ Copyright 2019, Elsevier B.V.

Considering their reactivity and biosafety in vivo, many magnetic NPs have been designed as acidity‐responsive NPs. After reaching the tumor sites, the magnetic NPs react with weak acids to exert their therapeutic effect. This prevents damage to normal tissues and enhances the degradation of magnetic NPs, further ensuring their biosafety in vivo.^[^ ^47^ ^]^ For example, Lee et al. designed a type of hierarchical tumor acidity‐responsive magnetic nanotheranostics (HTAMNs) for pH‐activated bimodal imaging and PDT (**Figure** **6**A). The HTAMNs were formed through the self‐assembly of chlorin e6 (Ce6), which was functionalized with a polypeptide ligand and superparamagnetic iron oxide NPs. After reaching tumor sites, HTAMNs were easily taken up by cancer cells because of the reversion of the surface charge caused by the acidic extracellular TME. As shown in Figure 6B, a Ce6 signal was discovered near the nucleus after incubation with HTAMNs at pH 6.5, which was opposite to that observed at pH 7.4. Moreover, almost no signal was detected inside cells treated with free Ce6 and non‐HTAMNs. These results demonstrated that the pH‐induced switching of their surface charge from negative to positive could effectively enhance the cellular internalization of HTAMNs. Meanwhile, the effect of PDT induced by HTAMNs was also demonstrated to depend on the change in pH (Figure 6C,D). Taken together, the in vivo results indicated that a weakly acidic TME could obviously promote the internalization, diagnostic sensitivity, and superior PDT effect of the HTAMNs.^[^ ^48^ ^]^

**Figure 7:**
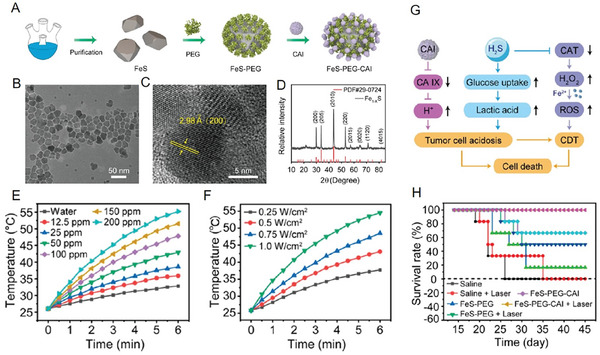
A) Schematic of the process of FeS‐PEG‐CAI NPs formation. B) TEM images of FeS NPs. C) HRTEM images of FeS NPs. D) XRD pattern of FeS NPs. E) Temperature changing curves of FeS‐PEG‐CAI NPs at different concentrations under irradiation using a 1064‐nm laser. F) Temperature changing curves of FeS‐PEG‐CAI NPs under irradiation using a 1064‐nm laser with different densities. G) Schematic illustration of the therapeutic mechanism of FeS‐PEG‐CAI NPs. H) Survival rate curves of mice that received different treatments. Reproduced with permission.^[^ ^51^ ^]^ Copyright 2022, American Chemical Society.

Tumor tissues present abnormal metabolic symbiosis, including weak acidity.^[^ ^49^ ^]^ It has the potential to disrupt the balance of the acidity in the TME for developing new cancer therapies.^[^ ^50^ ^]^ Recently, Hou et al. designed a type of carbonic anhydrase inhibitor (CAI)‐modified ferrous sulfide nanoparticles (FeS‐PEG‐CAI NPs). The results showed that FeS NPs with a mean diameter of ≈20 nm had good dispersion, suggesting an excellent uniformity with an irregular polygonal morphology (**Figure** **7**A–D). Surprisingly, the photothermal conversion efficiency of FeS NPs was as high as 56.51% under 1064 nm irradiation, which was much better than that observed for iron carbide NPs (Figure 7E,F). These results suggested FeS NPs as probable PTT agents. Notably, FeS‐PEG‐CAI NPs also possessed an acid‐responsive degradation capacity, and they could release some of their functional components, such as CAI, Fe^2+^, and H_2_S under acidic conditions. The generated CAI and H_2_S gas in the weak acidic TME could not only induce acidosis through the disruption of the intracellular metabolic symbiosis but also provide suitable conditions for Fe^2+^‐mediated Fenton reaction, which led to the production of toxic ·OH, promoting tumor cell death (Figure 7G). The results of animal experiments also indicated that this synergistic CDT/PTT/gas therapy prolonged the survival period of tumor‐bearing mice by inhibiting tumor growth (Figure 7H).

### 4.2 H_2_O_2_‐Responsive Theranostic Nanoplatforms

The high H_2_O_2_ content in the TME is often a key design point for the application of magnetic NPs in the field of cancer theranostics, such as CDT.^[^ ^52^ ^]^ Because of the limited penetration of conventional PDT into deep tissues, CDT is an alternative treatment to generate ·OH and ROS for eliminating cancer cells without using light excitation. Emerging nanotechnologies called nanozymes also perfectly conform to the working principle of CDT.^[^ ^53^ ^]^ Unlike natural enzymes, nanozymes, which are nanomaterials with enzyme‐like characteristics, have several unique advantages, such as high stability, low cost, and ease of mass production.^[^ ^54^ ^]^ In the early stages, many iron oxide NPs (e.g., Fe_3_O_4_) exhibited potential for their application as nanozymes in tumor diagnosis and treatment.^[^ ^55^ ^]^ For example, Shi et al. fabricated GOx‐Fe_3_O_4_@DMSNs nanocatalysts by encapsulating glucose oxidase (GOx) and ultrasmall Fe_3_O_4_ NPs in dendritic mesoporous silica NPs (DMSNs). The meticulous design of these nanocatalysts relied on the introduction of GOx, which depleted glucose to starve cancer cells and produced high concentrations of H_2_O_2_, which could continuously provide substrates for Fe_3_O_4_ NP‐mediated Fenton‐like reactions.^[^ ^55^ ^]^

**Figure 8:**
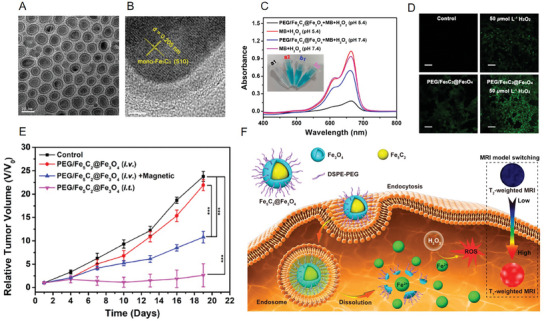
A) TEM image of Fe_5_C_2_@Fe_3_O_4_ NPs. B) HRTEM image of Fe_5_C_2_@Fe_3_O_4_ NPs. C) UV–vis absorption spectra and photos of the MB and H_2_O_2_ mixture after degradation by PEG/Fe_5_C_2_@Fe_3_O_4_ NPs at pH 5.4 and 7.4 for 24 h. D) Fluorescence images of DCFH‐DA labeled 4T1 cells treated with Fe_5_C_2_@Fe_3_O_4_ NPs under different incubation conditions. E) Tumor relative volume curve of tumor‐bearing mice that received different treatments. F) Schematic illustration of Fe_5_C_2_@Fe_3_O_4_ NPs for pH‐responsive Fe^2+^ release, ROS, and T_2_/T_1_ signal conversion. Reproduced with permission.^[^ ^11^ ^]^ Copyright 2019, American Chemical Society.

In recent years, based on the reported catalytic activity of iron carbide NPs, researchers have found that iron carbide NPs are also an option for nanozymes. Hou et al. performed extensive and systematic work in this field. In 2019, they designed and synthesized an ROS nanoreactor based on Fe_5_C_2_@Fe_3_O_4_ NPs with core–shell structures. The results of TEM/HRTEM showed that the core part consisted of Fe_5_C_2_ with a highly crystallized structure and the shell region comprised Fe_3_O_4_ amorphous entities with several tiny crystalline domains (**Figure** **8**A,B). Subsequently, the catalytic activity in vitro was measured (Figure 8C). Fe_5_C_2_@Fe_3_O_4_ NPs and H_2_O_2_ at pH 5.4 were incubated with an ROS indicator, i.e., methylene blue (MB); the results showed that Fe_5_C_2_@Fe_3_O_4_ NPs responded to the weak acidic conditions, and high H_2_O_2_ content of the TME and generated an abundant ∙OH and ROS, as indicated by the decreased absorbance of MB. This experiment proved that Fe_5_C_2_@Fe_3_O_4_ NPs could efficiently produce ROS when incubated with exogenous H_2_O_2_ (Figure 8D) at the cellular level and exert a significant inhibitory effect on tumor cell growth at the animal level (Figure 8E). Moreover, dual T_2_/T_1_ modal MRI mediated by this ROS reactor could also trigger the release of Fe^2+^, and the production of ROS was visualized, resulting in the supervision of the tumor treatment process (Figure 8F).^[^ ^11^ ^]^ To further enrich the biological functions of nanozymes, Hou et al. reported a multifunctional magnetic theranostics nanoprobe with enzyme‐like activity based on TME “unlocking” Ag_2_S@Fe_2_C‐DSPE‐PEG‐iRGD NPs. Among them, Ag_2_S played a role in NIR II imaging, whereas Fe_2_C acted as a PTT/CDT trigger. Modification with iRGD led to the active targeting of tumor cells, allowing these nanozymes to reside at the tumor sites for a longer period. In terms of theranostics, Ag_2_S@Fe_2_C‐DSPE‐PEG‐iRGD NPs combined with bevacizumab, which normalizes tumor blood vessels, exhibited an excellent tumor inhibition effect through PTT and CDT. Moreover, the entire treatment process was monitored via MRI/NIR II fluorescence imaging dual‐mode imaging.^[^ ^56^ ^]^

**Figure 9:**
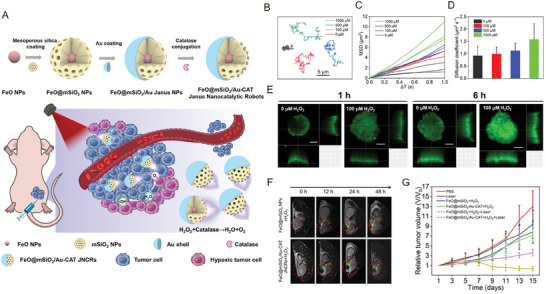
A) Schematic of the synthesis process and theranostic strategy of JNCRs. B) Representative tracking trajectories of JNCRs at 0, 100, 500, and 1000 µM H_2_O_2_. C) MSD plots of JNCRs at 0, 100, 500, and 1000 µM H_2_O_2_. D) Diffusion coefficients of JNCRs at 0, 100, 500, and 1000 µM H_2_O_2_. E) Images of a 4T1 tumor spheroid incubated with 5‐FAM‐labeled JNCRs at 0 and 100 µM H_2_O_2_ for 1 and 6 h. F) Representative in vivo T2‐weighted MR images of subcutaneous 4T1 tumors in mice that received different treatments. G) Tumor relative volume curve of tumor‐bearing mice that received different treatments. Reproduced with permission.^[^ ^57^ ^]^ Copyright 2023, American Chemical Society.

The responsiveness of magnetic NPs to H_2_O_2_ can be conducive to some emerging therapies; therefore, Hou et al. also endow magnetic NPs with a driving force in vivo based on this characteristic. To address the issue of insufficient tissue penetration of these functional magnetic NPs, self‐propelled Janus nanocatalytic robots (JNCRs) were designed. To prepare Janus FeO@mSiO_2_/Au NPs, Au NPs were sputtered onto the hemisphere of FeO@mSiO_2_‐NH_2_ NPs. Catalase (CAT) was then covalently conjugated onto the noncoated side of Janus NPs via a glutaraldehyde linker molecule (**Figure** **9**A). The movement trajectories of the single JNCRs are presented in Figure 9B. In contrast to the Brownian movement of JNCRs in the H_2_O solution, an expanded range of motion was caused by the reaction between CAT and the H_2_O_2_ solution. As shown in Figure 9C,D, the mean‐squared displacement (MSD) and effective diffusion coefficient of JNCRs were also analyzed, which further proved the effectiveness of the self‐propulsion of JNCRs. Additional results showed that JNCRs could better penetrate the tumor tissue in the presence of H_2_O_2_ (Figure 9E,F), eventually leading to a good tumor treatment effect (Figure 9G).^[^ ^57^ ^]^

### 4.3 Immune System‐Responsive Theranostic Nanoplatforms

Cancer immunotherapy is a dynamic and systemic treatment that regulates the activity of immune cells and enables them to attack cancer cells or prevent them from escaping.^[^ ^58^ ^]^ The present cancer immunotherapies include cytokine therapy,^[^ ^59^ ^]^ monoclonal antibody (mAb) therapy,^[^ ^60^ ^]^ immune checkpoint blockade (ICB),^[^ ^61^ ^]^ cancer vaccines,^[^ ^62^ ^]^ and adoptive cell transfer (ACT).^[^ ^63^ ^]^ With the development of new nanotechnologies, an increasing number of researchers aim to design nanomaterials that can respond to the immune system in vivo for immunomodulatory activation. Various magnetic NPs have been used in cancer immunotherapy. Iron‐based NPs greatly contribute to immunotherapy because of their intrinsic immunogenicity. Initially, iron‐based NPs were designed as artificial antigen‐presenting cells on the nanoscale dimension, to trigger antigen‐specific T cell proliferation.^[^ ^64^ ^]^ Subsequently, ovalbumin and magnetic Fe_3_O_4_ NPs were combined to develop nanovaccines. By activating the immune cells and producing cytokines, Fe_3_O_4_ NP‐based vaccines exhibited a straightforward immunogenicity.^[^ ^65^ ^]^ Moreover, magnetic NPs can also be used for the delivery of immune‐related drugs. Iron oxide–zinc oxide core–shell NPs were prepared to transport carcinoembryonic antigens. Dendritic cells (DCs) that capture targeted magnetic NPs and present antigens to T cells triggered specific T‐cell responses to generate a systemic immune response for killing tumor cells.^[^ ^66^ ^]^ Moreover, an external magnetic field can also trigger iron oxide NPs to direct different immune cells including DCs^[^ ^67^ ^]^ and T cells,^[^ ^68^ ^]^ toward tumor cells.

**Figure 10:**
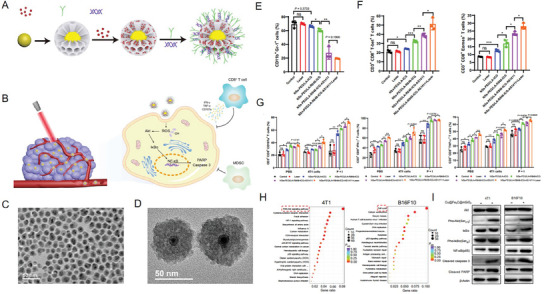
A) Schematic of the synthesis process of Cu@Fe_2_C@mSiO_2_‐R848‐ICG‐AS1411 NPs. B) Schematic of the treatment mechanism of Cu@Fe_2_C@mSiO_2_‐R848‐ICG‐AS1411 NPs. C) TEM images of Cu@Fe_2_C NPs. D) TEM images of Cu@Fe_2_C@mSiO_2_ NPs. E) Statistical analysis of the flow cytometry of CD11b^+^Gr‐1^+^ splenocytes of mice that received different treatments. F) Statistical analysis of the flow cytometry of CD3^+^CD8^+^T‐bet^+^ and CD3^+^CD8^+^Eomes^+^ splenocytes of mice that received different treatments. G) Statistical analysis of the flow cytometry of CD3^+^CD8^+^CD107a^+^, CD3^+^CD8^+^IFN‐ɣ^+^, and CD3^+^CD8^+^TNF‐ɑ^+^ splenocytes of mice that received different treatments. H) Gene ontology enrichment analysis of 4T1 and B16F10 cells incubated with Cu@Fe_2_C@mSiO_2_ NPs for 24 h. I) Western blotting of 4T1 and B16F10 cells incubated with Cu@Fe_2_C@mSiO_2_ NPs for 24 h. Reproduced with permission.^[^ ^69^ ^]^ Copyright 2022, American Association for the Advancement of Science.

Interestingly, iron carbide NPs have exhibited great potential in cancer immunotherapy. Hou et al. have developed a series of immune system‐responsive theranostic nanoprobes. Novel immunomodulatory nanozymes based on Cu@Fe_2_C@mSiO_2_‐R848‐ICG‐AS1411 were designed for real‐time visualization and synergistic cancer therapy.^[^ ^69^ ^]^ The design concept was as follows: Fe_2_C played the role of MRI, PTT, and CDT and the addition of copper ions further enhanced the photothermal conversion performance and peroxidase‐like activity of iron carbide NPs.^[^ ^70^ ^]^ Moreover, this synergistic cancer therapy could also generate massive cancer cell fragments as antigens, which was the first key step in the activation of the immune system. Coating the surface of Cu@Fe_2_C NPs with mesoporous silica combined with phase change materials (polyethylene glycol (PEG)/lauric acid) further realized the loading and release of immune agonists (R848).^[^ ^71^ ^]^ Moreover, ICG and the nucleolin‐specific aptamer AS1411^[^ ^72^ ^]^ were attached to the surface of immunomodulatory nanozymes for NIR II imaging and active targeting of tumor cells, respectively (**Figure** **10**A). Notably, the antitumor mechanism of this immunomodulatory nanozyme system was systematically explored, including specific immune responses and intrinsic molecular signaling pathways (Figure 10B). The results presented in Figure 10C,D indicated the good dispersibility of Cu@Fe_2_C NPs and Cu@Fe_2_C@mSiO_2_ NPs, which lay a foundation for subsequent in vivo experiments. In addition to the excellent PTT and CDT effects of Cu@Fe_2_C@mSiO_2_‐R848‐ICG‐AS1411 NPs, another important discovery was their activation effect on the immune system. These results demonstrated that Cu@Fe_2_C@mSiO_2_‐R848‐ICG‐AS1411 NPs substantially downregulated the proportion of myeloid‐derived suppressor cells as a kind of immunosuppressive cells to alleviate the immunosuppression of the TME (Figure 10E). Next, by intrinsically upregulating the expression of T‐bet^[^ ^73^ ^]^ and Eomesodermin (Eomes),^[^ ^74^ ^]^ the key transcription effectors of CD8^+^ T cells, Cu@Fe_2_C@mSiO_2_‐R848‐ICG‐AS1411 NPs induced the greatest degree of CD8^+^ T cells activation (Figure 10F), and further enhanced the function of CD8^+^ T cells by increasing the secretion of cytokines (Figure 10G). In terms of molecular signaling pathways, a gene ontology enrichment analysis, which identified several key signaling pathways that were affected by Cu@Fe_2_C@mSiO_2_ NPs has been found (Figure 10H). Subsequently, these results were validated at the cellular level. The results indicated that Cu@Fe_2_C@mSiO_2_ NPs activated the Akt and IκBɑ kinases, then increased the expression of the NF‐κB(p65) transcription factor, and consequently upregulated the expression of cleaved‐caspase 3 and cleaved‐PARP, resulting in tumor cell apoptosis (Figure 10I). This study provided important therapeutic strategies and theoretical basis for clinical medicine.

In addition to influencing T cells, Hou et al. found that iron‐based NPs also have an effect on DCs and macrophages. A relatively simple catalytic immune activator Au–Fe/Fe_3_O_4_@PAA‐ICG‐AS1411 NP was designed and prepared. The results showed that Au–Fe/Fe_3_O_4_ NPs had a better catalytic activity than Au–Fe_2_C NPs, which might also explain the higher immune response induced by Au–Fe/Fe_3_O_4_ NPs versus Au–Fe_2_C NPs. In particular, these catalytic immune activators not only facilitated DC maturation but also polarized the macrophages from the M2‐like state to the M1‐like state, leading to the infiltration of a greater number of T cells into the TME. On the other hand, these catalytic immune activators could also indirectly enhance the immune reaction by modulating the secretion of several immune‐related cytokines by cancer cells. As a result, the suppressive TME of triple‐negative breast cancer (TNBC) has been extremely ameliorated, together with the reprogramming of the immunoecology of TNBC from the “cold” state to the “hot” state.^[^ ^26^ ^]^

## 5 Conclusions and Outlook

The ability to respond to exogenous or endogenous stimuli is a major consideration in the future design of magnetic nanomaterials, which can make them smarter and safer. Although some conventional iron oxide NPs have been developed, because of limitations in the intrinsic properties of these magnetic nanomaterials, researchers are extremely eager to produce new types of magnetic nanomaterials. Iron carbide NPs with multiple excellent physical and chemical properties, may further expand the application of magnetic nanomaterials in the biomedical field. Higher expectations are always accompanied by more stringent requirements. One of the most important reasons for the restrictions on the clinical translation of iron carbide NPs is their biosafety in vivo, including degradation, metabolism, and ethical issues. All of these factors must be key considerations for the future application of iron carbide NPs in biomedicine. Large‐scale manufacturing is another limiting factor of iron carbide NPs, which is highly demanding regarding the selection of synthesis methods. In addition to the standardization and stability of the synthesis process, the economic benefits should also be considered.

Regarding the responsiveness of magnetic NPs to exogenous or endogenous stimuli, their design strategy for tumor diagnosis and treatment needs to be continuously innovated. Traditional therapies, such as PTT, PDT, and CDT, have their own limitations, because they cannot meet the needs of dealing with the changeable and complex TME. Hence, nanotechnology‐mediated cancer immunotherapy and burgeoning micro/nanorobots may become a vigorous research area. Therefore, the identification of the most suitable driving force in response to exogenous or endogenous stimuli is the focus of micro/nanorobot designing, which further enhances the implementation of the quantitatively controlled motion. Finally, the design of micro/nanorobots should also consider the pre‐clinical feasibility and clinical demand.

## Conflict of Interest

The authors declare no conflict of interest.
